# Supplementary material for: Relationship between cigarette smoking and blood pressure in adults in Nepal: A population-based cross-sectional study
Source: PLOS Glob Public Health. 2021 Nov 9;1(11):e0000045. doi: 10.1371/journal.pgph.0000045 (PMC10022357; doi:10.1371/journal.pgph.0000045)
Supplement: Attachment — Submitted filename: Response to Reviewers_PGPH-D-21-00510.docx [file pgph.0000045.s001.docx]

Dr Hilary Wallace

Adjunct Senior Research Fellow

University of Western Australia

School of Population and Global Health

Palash Chandra Banik, MPhil
Academic Editor
PLOS Global Public Health

15 September 2021

Dear Mr Banik,

*Re: Revisions to PGPH-D-21-00510: Relationship between cigarette smoking and blood pressure in adults in Nepal: A population-based cross-sectional study*

Please find our changes listed below. We have made the same modifications to the manuscript itself. All authors have approved the revisions. We thank the reviewers for their constructive comments and feedback. Thank you for your consideration of our revisions.

Yours sincerely,

Hilary Wallace (on behalf of the authorship team)

**Response to Reviewers Comments (in red)**

**Reviewer 1** – No comments to address

**Reviewer 2** –

ABSTRACT:
Commencement of the abstract was good. But conclusion is too hazy and in some points i failed to understand what the authors actually intended to say. I think a conclusion should be ln such pattern what the readers can adopt in a single reading. So, i will request the authors to work on ABSTREACT and to make it more succinate.

We have clarified the conclusion of the abstract and the penultimate sentence:

“The lower BP in female cigarette smokers in Nepal may be explained by the physiological effect of daily cigarette smoking *per se* in women, or unmeasured confounders associated with a traditional lifestyle that may lower BP (for example, diet and physical activity). In this nationally representative survey, daily cigarette smoking was not associated with increased blood pressure in males or females in Nepal. ~~The finding that cigarette smoking is not associated with increased BP in Nepali adults up to 49 years in a nationally representative survey contributes to the understanding of the relationship between these two major risk factors for cardiovascular disease but does not change public health advice on smoking cessation.~~”

BODY of the STUDY:
Introduction and Methods are well written and clear.
Few queries-
1. Is ciigerrete smoking is so common in women of Nepal? If not, then the title should be corrected using tobacco smoking , rather than cigerrete smoking. It is my opinion, authors can differ and defend.

This study was concerned with cigarette smoking specifically (manufactured and hand rolled). Cigarette smoking is the most common form of tobacco smoking by women in Nepal. We have added the following sentence in the Introduction:

“Cigarette smoking is the most common form of tobacco smoking by men and women in Nepal (24).”

2. BP measurement: Three times BP measurements have been taken within 5 minutes interval. Does it create any variation? And have you found any reference or guideline for measuring BP in 5 minutes3. interval? If yes, then please mention and clarify your thoughts.

The blood pressure measurement protocol used in the Demographic and Health Survey (DHS) is standardised across surveys conducted in many countries, as defined in the standard DHS biomarker collection protocol. Three readings are made at a minimum of 5-minute intervals. The average of the second and third measurements are used. We have added the following phrase in the Methods (Variables):

“The first measurement was discarded and the average of the second and third measurements was recorded as the final reading and recorded as a continuous variable (mm Hg) according to the standard DHS biomarker collection protocol (24).”

3. Why does the authors keep differences on number of cigerrete smoking for men and women? What is the exact reason here? If any specific reason with reference is available, please mention.

The reason we presented the numbers of cigarettes smoked by men and women is for descriptive purposes (smoking heaviness). We have now included the following phrase in the Results:

“A smaller proportion of women (4.8%) smoked cigarettes daily than men (18.9%), and the same proportion (27%) of women and men smokers were moderate to heavy smokers (10 cigarettes or more per day [17]).”

We already acknowledge in the Discussion that a limitation of the study was that we were unable to undertake sub-group analysis by smoking level due to a lack of statistical power.

“Other limitations include a lack of statistical power to undertake sub-group analysis by level of smoking.”

5. Have the authors included SLT in their studies? Again this tern cigerrete smoking is creating a confusion here. But i found , no where it is mentioned properly. Please clarify this.

The inclusion criterion for smokers was daily cigarette smoking (hand-rolled or manufactured). We did not exclude people who used smokeless tobacco from the current smoker group or the non-smoker group. We have added the following sentence to the limitations section of the Discussion.

“We also did not exclude individuals who had smoked or consumed alcohol or caffeine within 30 minutes before the BP readings, or who were users of smokeless tobacco, which may be a source of confounding.”

6. As proper physical activity measurement guidelines, questionnaires are available , so why you considered agriculture activity only as a proxy of physical activity? Is there any specific reason.

This study used secondary data from the Nepal 2016 Demographic and Health Survey (DHS). The DHS survey unfortunately does not collect any data on physical activity; for this reason, we used “works in agriculture” as a proxy variable.

Lastly discussion is also well written. But again problem faced to understand the conclusion of the study. Somehow, it is not that clear as like the whole study. So, i wish authors will work on that if they feel.

We have attempted to make the message of the conclusion clearer by rearranging the sentences.

**“Conclusions**

This study describes the association between cigarette smoking and blood pressure in adults in Nepal aged 18-49 years. Our finding that daily cigarette smoking was not associated with increased BP in men or women in this population contributes to the understanding of the relationship between these two major risk factors for cardiovascular disease in populations that share characteristics with Nepal. However, the results of this study should not be used to influence the public health campaigns on smoking cessation as cigarette smoking is a strong independent risk factor for cardiovascular disease. Future research employing longitudinal studies, the use of ambulatory BP monitoring, adjusting for additional confounders, and studies on arterial stiffness and central blood pressure (12) may provide further insight into of the effect of smoking on BP. ~~The results of this study should not be used to influence the public health campaigns on smoking cessation as cigarette smoking is a strong independent risk factor of cardiovascular disease.~~”
